# Paediatric Catatonia as a First Presentation of Autism: A Case Report

**DOI:** 10.1192/bjo.2024.673

**Published:** 2024-08-01

**Authors:** Vinay Mandagere, Helen Stephens, Kitty Kwan, Jatinder Singh, Paramala Santosh

**Affiliations:** 1 Bristol Royal Infirmary, Bristol, United Kingdom; 2 Avon and Wiltshire Mental Health Parternship Trust, Bristol, United Kingdom; 3 Department of Child and Adolescent Psychiatry, King's College London, London, United Kingdom; 4 Centre for Interventional Paediatric Psychopharmacology and Rare Diseases (CIPPRD), Maudsley Hospital, London, United Kingdom

## Abstract

**Aims:**

Catatonia is a rare neuropsychiatric syndrome in children. It is characterised by mutism, stupor, posturing, negativism, and rigidity. Historically, catatonia was associated only with psychosis, however catatonic symptoms are being recognised as more prevalent in people with Autism Spectrum Disorder (ASD). Our case report highlights the importance of investigating the potential underlying psychopathology and/or neurodevelopmental condition as this may guide management.

**Methods:**

We present a case of a boy in early adolescence who was admitted to the Emergency Department for abnormal slowing of movement and stuttered speech. He described losing all interest in his hobbies, lying down for long periods of time, sometimes being unresponsive and ‘freezing’ in place. On examination, his symptoms were consistent with catatonia: mutism, grimacing, abnormal gait, and ambitendency were all present. He was investigated extensively to rule out medical and neurological causes, all of which were normal. He was assessed and managed by the Centre for Interventional Paediatric Psychopharmacology and Rare Diseases (CIPPRD). After appropriate treatment, he was discharged from the hospital and was managed jointly by CIPPRD and the local Child and Adolescent Mental Health Service (CAMHS). This assessment revealed that the presentation of catatonia occurred during a depressive episode on a background of ASD and underlying Intellectual Disability. He was prescribed fluoxetine as opposed to benzodiazepines or antipsychotics, which led to the catatonic symptoms receding. The neurodevelopmental review revealed that his pattern of social communication and speech after catatonia improvement was consistent with ASD, which was then formally diagnosed.

**Results:**

Untreated catatonia can be fatal. Early diagnosis and management are crucial to avoiding complications. Our case report highlights the challenge of treating paediatric catatonia and the diagnostic and therapeutic importance of understanding underlying psychopathology to decide treatment. Studies have shown that in this population, assessing and treating the underlying psychopathology as opposed to sole use of the lorazepam is essential.

**Conclusion:**

Catatonia in paediatric and adolescent populations may be a first presentation of emotional and behavioural problems underlying autism spectrum disorder (ASD). When treating catatonia, consideration of the underlying psychopathology may warrant alternative pharmacological treatments to the traditional lorazepam challenge test and antipsychotics. The course of catatonia and associated comorbid affective and/or psychotic disorders may fluctuate with environment and therefore a biopsychosocial therapeutic model is warranted.

